# Recent progress of ultra-narrow-bandgap polymer donors for NIR-absorbing organic solar cells

**DOI:** 10.1039/d1na00245g

**Published:** 2021-06-09

**Authors:** Dae-Hee Lim, Jong-Woon Ha, Hyosung Choi, Sung Cheol Yoon, Bo Ram Lee, Seo-Jin Ko

**Affiliations:** Department of Physics, Pukyong National University 45 Yongso-ro, Nam-Gu Busan 48513 South Korea brlee@pknu.ac.kr; Division of Advanced Materials, Korea Research Institute of Chemical Technology (KRICT) Daejeon 34114 South Korea sjko927@krict.re.kr; Department of Chemistry, Institute of Nano Science & Technology, Research Institute for Natural Sciences, Hanyang University 04763 Seoul South Korea

## Abstract

Solution-processed near-infrared (NIR)-absorbing organic solar cells (OSCs) have been explored worldwide because of their potential as donor:acceptor bulk heterojunction (BHJ) blends. In addition, NIR-absorbing OSCs have attracted attention as high specialty equipment in next-generation optoelectronic devices, such as semitransparent solar cells and NIR photodetectors, owing to their feasibility for real-time commercial application in industry. With the introduction of NIR-absorbing non-fullerene acceptors (NFAs), the value of OSCs has been increasing while organic donor materials capable of absorbing light in the NIR region have not been actively studied yet compared to NIR-absorbing acceptor materials. Therefore, we present an overall understanding of NIR donors.

## Abstract 2

This review provides a comprehensive understanding of the chemical structure and overall development trend for solution-processable NIR-absorbing ultra-narrow-bandgap (UNBG; below 1.5 eV) polymer donors used in organic solar cells.

## Graphical abstract

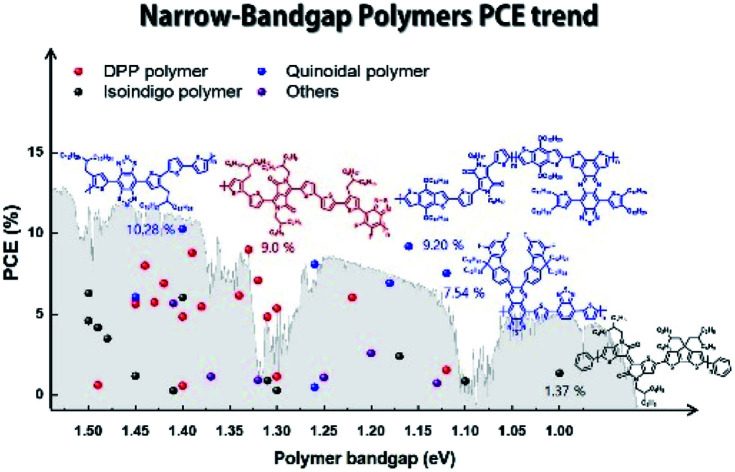

## 1 Introduction

The development of clean and sustainable energy is becoming essential worldwide, as increased urbanization and industrialization are accelerating expectations and demand for future energy resources. Among various renewable energy resources, such as solar and wind energy, biomass, and fuel cells, and in particular green energy sources, research on generating electrical energy from natural light through photocurrent conversion has progressed rapidly over the past few decades with enormous research efforts. Organic solar cells (OSCs) are advantageous for solar-energy applications because they can be used in terms of various perspectives based on the unique advantages of organic semiconductors, such as processability in solution, light weight, low cost, flexibility, semitransparency, and suitability for large-scale roll-to-roll processing.^1,2^

**Fig. 1:**
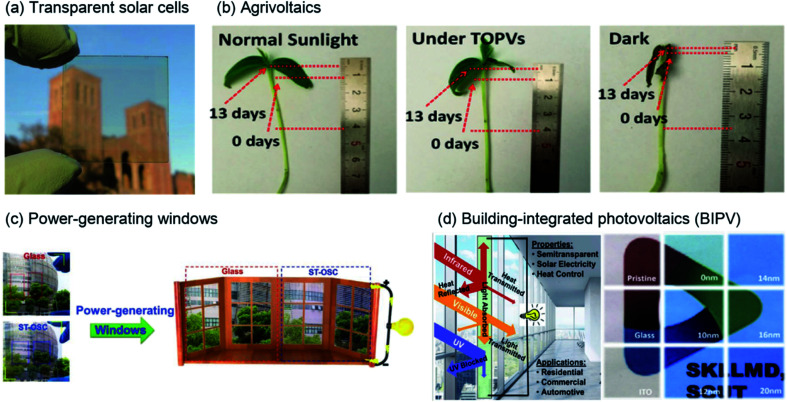
Illustration of the various applications of NIR-absorbing solar cells. (a) Visibly transparent polymer solar cell.^11^ Copyright 2012 American Chemical Society. (b) Potential agricultural application of flexible transparent organic solar cells (OSCs); length change of mung beans under different light conditions.^12^ Copyright 2019 American Chemical Society. (c) Digital photographs of a doctor-blade-coated J71:PTB7-Th:IHIC-based ternary ST-OSC and glass counterparts. Right: used as glass applied to windows.^10^ Copyright 2019, Wiley-VCH. (d) Schematic representation of a ST-OSC for power generation and heat-insulation applications. Reproduced with permission.^13^ 2018, Elsevier Inc.

Recently, semi-transparent (or visibly transparent) solar cells using NIR regions have gained attention for various applications, including in existing windows, rooves, vehicles, mobile electronic devices, and sensors, owing to their advantage of semitransparency.^3–10^ As shown in Fig. 1(a), Chen *et al.* realized high-performance, visibly transparent solar cells by using a diketopyrrolopyrrole (DPP)-based polymer to harvest solar energy from the NIR region.^11^ In addition, the use of transparent solar cells can extend beyond simple solar-cell applications. When energy is generated from light in the NIR region, the essential wavelength region of sunlight used by animals and plants remains; thus, a synergistic effect is created among the environment, life, and energy production. As shown in Fig. 1(b), Liu *et al.* performed plant (mung) growth under normal sunlight and transparent solar cells. They selected the donor polymer PTB7-Th and the acceptor IEICO-4F as photoactive materials for photocurrent production. Under transparent solar cells or normal sunlight, the final lengths of mung were comparatively similar.^12^ The penetrated visible light is utilised for photosynthesis in plants, indicating the potential for constructing self-powered greenhouses using transparent solar cells. Moreover, for human beings, transparent solar cells can be used for not only generating power from sunlight but also for solar shading and heat insulation employed in power-generating windows and building-integrated photovoltaics, as shown in Fig. 1(c) and (d), respectively, and reported by Zhang *et al.* and Sun *et al.*^10,13^

Therefore, to realise these applications, photoactive materials that absorb light from the NIR region must be developed. To absorb light in the NIR region, both donor and acceptor materials in a bulk heterojunction system must have a smaller band gap than the energy range of visible wavelengths (380–780 nm) for photocurrent generation. In the development of acceptor materials, many narrow-band gap non-fullerene acceptors (NFAs) have been reported, and their power conversion efficiency (PCE) is continuously being updated.^14–19^ However, studies have reported a PCE of <10% in the existing narrow-band gap polymer donors, and their efficiency tends to decrease as the band gap decreases. To achieve transparency, a material with an extremely small band gap (over the 830 nm band gap edge and below the band gap energy of 1.5 eV) is necessary to minimize absorption in the visible region. Thus, significant research and development of donor materials is required.

**Fig. 2:**
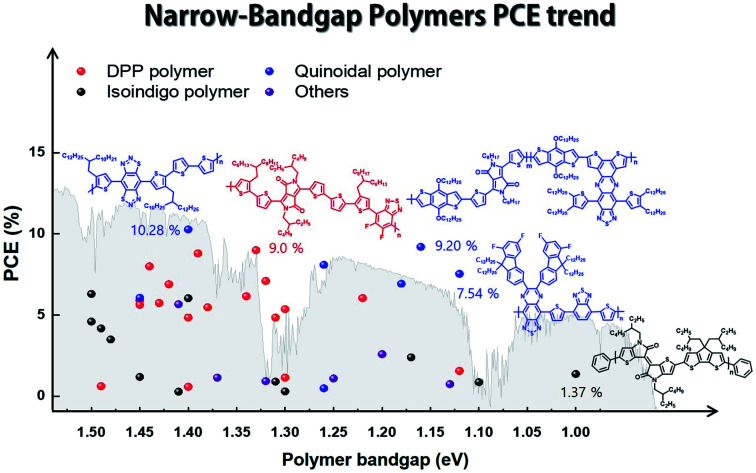
Band gap and power conversion efficiency trends in narrow-band gap polymers for organic solar cells.

In this review, we focus on solution-processed NIR-absorbing ultra-narrow-band gap (UNBG; below 1.5 eV) polymer donors. These polymers are usually called UNBG materials and can be applied to NIR OSCs. We investigated polymers based on their optical band gap (*E*_g_); thus, all polymers included in this review have absorption edges over 830 nm. In this paper, the discussed UNBG polymers are organized from past high-performance structures to the most recent achievements and trends. The representative material trends are shown in Fig. 2. In terms of chemical structure, the narrow band gap is affected by the strong electron-withdrawing group within the polymer chain; thus, these polymers are discussed and summarized according to the electron-withdrawing group type. Finally, we conclude with a discussion on the chemical structure and overall development trend of narrow-band gap polymers.

## 2 Narrow-band gap polymer donor materials for NIR organic solar cells

### 2.1 Molecular design strategies for UNBG polymer donors

**Fig. 3:**
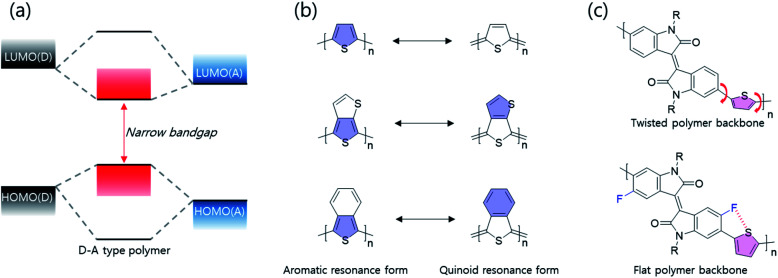
Illustration of the molecular design strategies for NIR-absorbing polymer donors. (a) Band gap narrowing mechanism of a D–A type polymer. (b) Aromatic resonance form verse the quinoidal resonance form. (c) Schematic representation of the strategy for the enhancement of coplanarity along the polymer main chain.

To effectively reduce the optical band gap to the NIR range, systematic synthesis strategies are as follows; first of all, donor–accepter (D–A)-type polymer donors have the ability to effectively adjust intramolecular charge transfer (ICT) effect between electron-donating and -accepting building blocks, thereby reducing the optical band gap as shown in Fig. 3(a). According to molecular orbital theory, the highest occupied molecular orbital (HOMO)/the lowest unoccupied molecular orbital (LUMO) of a D–A type polymer donor originated from the combination of HOMOs/LUMOs of the electron-donating and -accepting building blocks. Thus, D–A type polymers involving orbital hybridization and electron redistribution between electron-donating and -accepting building blocks show a narrow band gap. In particular, the combination of strong electron-donating and -accepting building blocks, such as diketopyrrolopyrrole (DPP) and isoindigo, is able to reduce the optical bandgap more to the NIR range because the ICT effect is proportional to the electron-donating/accepting strengths. Secondly, the electron-deficient quinoid type has been used in NIR semiconducting materials, as shown in Fig. 3(b). In particular, a fused molecular structure that can stabilize a quinoidal resonance form is more effective in narrowing the band gap than a common thiophene derivative.^20,21^ Compared with aromatic resonance molecules, the quinoidal resonance molecule is well known to effectively reduce the optical band gap because conjugate segments are linked by energetic double bonds, resulting in stabilization of the quinoidal resonance form compared with the aromatic resonance form. In addition to those described above, various factors including molecular coplanarity, inter/intramolecular interactions, and substitution of the electron-withdrawing unit onto the polymer backbone should be considered. As shown in Fig. 3(c), such a fluoro-atom introduced by fluorination into the backbone can form the S–F interactions with the adjacent thiophene unit, which can induce backbone planarization and enhance intra- and inter-molecular interactions. The progress of research on band gap narrowing for polymer donors including all these strategies is summarized and discussed according to the electron-withdrawing group type.

### 2.2 DPP-based narrow-band gap polymers

**Fig. 4:**
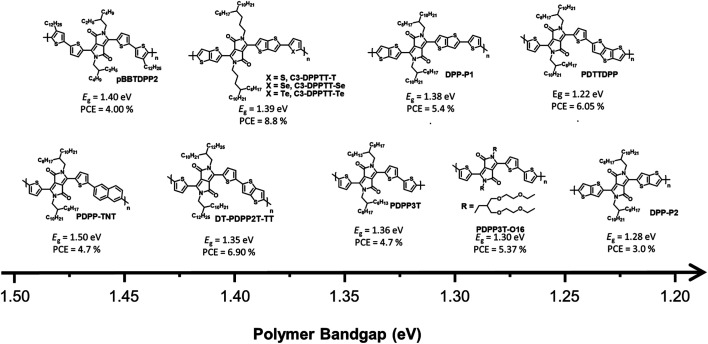
Chemical structures of DPP-based polymers used in the past (∼2019).

**Fig. 5:**
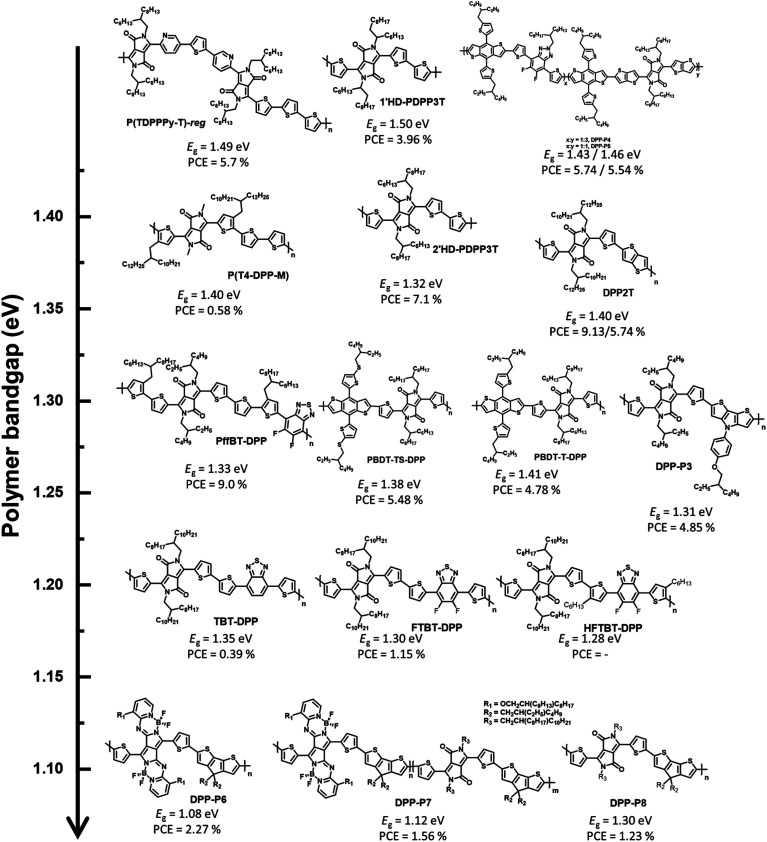
Chemical structures of the most recent progress in DPP-based polymers (after 2019).

DPP-based conjugated polymers and small molecules have been widely researched in the field of organic electronics for applications, such as OSCs, organic field-effect transistors, and organic photodiodes owing to the superior properties, including strong electron-withdrawing characteristics, planar geometric structures, and high hole/electron mobilities.^22–25^ Among semiconducting materials for NIR-absorbing OSCs, D–A type DPP-based conjugated polymers are promising candidates because they can produce strong intra/intermolecular charge transport effects between an electron-donating building block and DPP, resulting in a UNBG up to 1.22 eV. Among the many reported NIR-absorbing polymer donors, we focused on the most recent progress after a review in 2019 by Xie *et al.*^26^ Thus, this section deals with the understanding and analysis of previous DPP-based polymer donors (∼2019) as well as the latest DPP-based polymer donors (2019–present) for NIR-absorbing OSCs. Herein, we focused on UNBG DPP-based polymer donors with absorption edges over 830 nm (*E*_g_ < 1.50 eV). The summarized NIR-absorbing molecular structures, comprising DPP, depending on the optical band gap are shown in Fig. 4 and 5, and the optical, electrochemical, and photovoltaic performances of the polymer donors are summarized in Tables 1 and 2.

**Table 1:** Summary of the optical, electrochemical, and photovoltaic performances of past DPP-polymers

Materials	*E* ^opt^_g_ [eV]	*E*_LUMO_/*E*_HOMO_	Acceptor	*V*_OC_ [V]	*J*_SC_ [mA cm^−2^]	FF [%]	PCE [%]	*μ*_h_ [cm^2^ V^−1^ s^−1^]	*μ*_e_ [cm^2^ V^−1^ s^−1^]	Ref.
pBBTDPP2	1.40	—	PC_71_BM	0.61	11.5	0.58	4.0	—	—	27
PDPP-TNT	1.50	−3.79/−5.29	PC_71_BM	0.76	11.8	0.52	4.7	—	—	28
PDPP3T	1.36	−3.61/−5.17	PC_71_BM	0.65	11.8	0.60	4.7	—	—	29
DT-PDPP2T-TT	1.35	−3.68/−5.10	PC_71_BM	0.66	14.8	0.70	6.9	—	—	30
PDTTDPP	1.22	−3.80/−5.19	PC_71_BM	0.66	13.7	0.66	6.05	5.33 × 10^−3^	—	31
DPP-P1	1.38	−3.68/−5.06	PC_71_BM	0.58	15.0	0.61	5.4	—	—	32
DPP-P2	1.28	−3.76/−5.04	PC_71_BM	0.57	8.9	059	3.0	—	—	
C3-DPPTT-T	1.39	−3.69/−5.08	PC_71_BM	0.57	23.5	0.66	8.8	—	—	33
C3-DPPTT-Se	1.37	−3.70/−5.07	PC_71_BM	0.56	21.5	0.63	7.6	—	—	
C3-DPPTT-Te	1.32	−3.73/−5.05	PC_71_BM	0.52	21.7	0.63	7.1	—	—	
PDPP3T-O14	1.28	−3.65/−5.13	PC_71_BM	0.50	16.42	0.55	4.52	4.14 × 10^−3^	—	34
PDPP3T-O16	1.30	−3.61/−5.09	PC_71_BM	0.57	14.30	0.66	5.37	2.53 × 10^−3^	—	
PDPP3T-O20	1.33	−3.60/−5.21	PC_71_BM	0.56	4.82	0.44	1.20	—	—	
PDPP3T-C20	1.34	−3.50/−5.20	PC_71_BM	0.68	6.45	0.68	3.00	1.55 × 10^−3^	−*z*	

**Table 2:** Summary of the optical, electrochemical, and photovoltaic performances of recent DPP polymers

Materials	*E* ^opt^_g_ [eV]	*E*_LUMO_/*E*_HOMO_	Acceptor	*V*_OC_ [V]	*J*_SC_ [mA cm^−2^]	FF [%]	PCE [%]	*μ*_h_ [cm^2^ V^−1^ s^−1^]	*μ*_e_ [cm^2^ V^−1^ s^−1^]	Ref.
1′HD-PDPP3T	1.50	−3.13/−5.07	PC_71_BM	0.88	8.00	0.56	3.96	1.6 × 10^−3^	—	43
2′HD-PDPP3T	1.32	−3.13/−4.93	PC_71_BM	0.67	15.4	0.69	7.1	2.6 × 10^−3^	—	
PBDT-TS-DPP	1.38	−3.91/−5.29	PC_71_BM	0.78	11.52	0.61	5.48	2.37 × 10^−4^	1.30 × 10^−4^	44
PBDT-TDPP	1.41	−3.86/−5.27	PC_71_BM	0.74	9.46	68.30	4.78	2.40 × 10^−4^	1.04 × 10^−4^	
DPP-P3	1.31	−3.89/−5.20	PC_61_BM	0.79	12.82	0.48	4.85	—	—	45
P(T4-DPP-M)	1.40	−4.00/−5.40	PC_71_BM	0.56	16.4	0.58	5.4	—	—	46
			ITIC	0.69	6.7	0.46	2.2	—	—	
DPP2T	1.40	−3.70/−5.10	IEICO-4F	0.75	16.85	0.72	9.13	4.08 × 10^−3^	1.07 × 10^−3^	47
			IEICO-4F^a^	0.75	10.61	0.69	5.74	—	—	
P(TDPPPy-T)-*ran*	1.49	−3.20/−5.16	PC_71_BM	0.85	11.1	0.61	5.7	3.3 × 10^−6^	—	49
P(TDPPPy-T)-*reg*	1.49	−3.20/−5.19	PC_71_BM	0.87	11.8	0.62	6.4	8.4 × 10^−4^	—	
DPP-P4	1.43	−3.60/−5.28	PC_71_BM	0.62	15.20	0.60	5.74	1.3 × 10^−3^	4.91 × 10^−4^	59
			IEICO-4F	0.62	15.09	0.55	5.24	5.55 × 10^−4^	4.37 × 10^−4^	
DPP-P5	1.46	−3.57/−5.20	PC_71_BM	0.64	13.27	0.66	5.54	2.23 × 10^−4^	6.76 × 10^−5^	
			IEICO-4F	0.63	15.90	0.52	5.49	2.71 × 10^−4^	5.59 × 10^−5^	
PffBT-DPP	1.33	−3.56/−5.66	PC_71_BM	0.74	12.5	0.74	6.8	5.3 × 10^−4^	3.2 × 10^−4^	60
			MeIC	0.78	4.5	0.58	2.0	7.1 × 10^−4^	2.2 × 10^−4^	
			PC_71_BM:MeIC	0.76	16.1	0.73	9.0	6.2 × 10^−4^	4.0 × 10^−4^	
TBT-DPP	1.35	−3.42/−5.32	PC_71_BM	0.31	3.82	0.32	0.39	—	—	61
FTBT-DPP	1.30	−3.37/−5.35	PC_71_BM	0.63	3.31	0.54	1.15	—	—	
HFTBT-DPP	1.28	−3.45/−5.22	—	—	—	—	—	—	—	
DPP-P6	1.08	−4.11/−5.19	PC_71_BM	0.55	8.52	0.48	2.27	—	—	62
DPP-P7	1.12	−4.04/−5.16	PC_71_BM	0.54	6.25	0.46	1.56	—	—	
DPP-P8	1.30	−3.87/−5.17	PC_71_BM	0.59	3.36	0.62	1.23	—	—	

a Photovoltaic performances of semitransparent OSCs.

Generally, most previous DPP-based polymer donors (∼2019) were of the D–A type, and most of the electron acceptors in the corresponding OSCs were limited to fullerene-based derivatives. In 2008, Janssen *et al.* reported a DPP-based NIR-absorbing material (pBBTDPP2) that absorbed wavelengths up to 900 nm with the optical band gap of 1.40 eV. The OSC combining pBBTDPP2 and the fullerene derivative (PC_71_BM) exhibited the maximum PCE of 4.0%.^27^ Various DPP-based D–A-type polymer donors have been reported and studied since the development of pBBTDPP2. In particular, typical polymer donors constituting DPP and simple electron-donating building blocks such as naphthalene, thiophene, and thieno[3,2-*b*]thiophene have been used to produce PDPP-TNT, PDPP3T, and DT-PDPP2T-TT, respectively.^28–30^ Compared with PDPP3T and DT-PDPP2T-TT, PDPP-TNT has a relatively wide band gap (*E*_g_ = 1.50 eV) because naphthalene is a weaker electron-donating building block, which could lead to a weak intramolecular charge transfer (ICT) effect between DPP and naphthalene. Consequently, the OSC based on PDPP-TNT and PC_71_BM exhibited a PCE of 4.7% with the short-circuit current (*J*_SC_) of 11.8 mA cm^−2^. The strong ICT effect between DPP and thiophene or thieno[3,2-*b*]thiophene effectively generated narrow bandgaps of 1.36 eV for PDPP3T and 1.35 eV for DT-PDPP2T-TT, respectively. In particular, the OSC based on DT-PDPP2T-TT and PC_71_BM exhibited a maximum PCE of 6.9% with a significantly improved *J*_SC_ of 14.8 mA cm^−2^, which is related to the broad absorption range and high hole-carrier mobility of DT-PDPP2T-TT. Jo *et al.* introduced dithienothiophene (DTT) as a strong electron-donating building block to produce PDTTDPP, which could harvest a broad absorption range up to 1015 nm with a significantly narrow band gap (1.22 eV) because of the strong ICT effect between DPP and DTT as well as the extension of π-conjugation.^31^ Moreover, strong π–π intermolecular interactions have been observed in PDTTDPP, which are related to the improved charge carrier mobility. As a result, the optimum OSC based on PDTTDPP:PC_71_BM exhibited a PCE of 6.05%.

The π-bridges of DPP provide an effective strategy and have been extensively studied to modulate the optical band gap and frontier molecular orbital energy levels as well as π–π intermolecular stacking.^35–38^ McCulloch *et al.* reported thieno[3,2-*b*]thiophene-flanked DPP derivatives instead of thiophene as a π-bridge to extend π-conjugation, enhance polymer planarity, and promote charge carrier mobility.^32^ The corresponding polymer donor was polymerized with thiophene and thieno[3,2-*b*]thiophene-flanked DPP to produce DPP-P1, and a homopolymer (DPP-P2) was developed. DPP-P2 displayed a significantly broad absorption range with a narrow optical band gap (1.28 eV), while the optical band gap of DPP-P1 was 1.38 eV. The OSC based on DPP-P1 and PC_71_BM exhibited a higher PCE of 5.4% despite a narrow absorption range compared to DPP-P2; however, the PCE of DPP-P2:PC_71_BM was 3.0% with a reduced *J*_SC_ value of 8.9 mA cm^−2^ owing to the unfavourable morphology observed in the DPP-P2 film. Ashraf *et al.* demonstrated a chalcogenophene substitution strategy for a π-bridge in DPP-based polymers to effectively induce a narrow band gap.^33^ With increasing chalcogen atomic size, the optical band gaps of the desired polymers gradually narrowed owing to the stabilization of the lowest unoccupied molecular orbital (LUMO) energy level, high polarizability, and strong intermolecular interactions between molecules. Polymers containing selenophene and telluophene as π-bridges showed slightly narrower band gaps of 1.37 and 1.32 eV for C3-DPPTT-Se and C3-DPPTT-Te, respectively, than the reference polymer (C3-DPPTT-T, *E*_g_ = 1.39 eV). OSCs based on C3-DPPTT-Se:PC_71_BM and C3-DPPTT-Te:PC_71_BM exhibited moderate PCEs of 7.6% and 7.1%, respectively, while the highest PCE was achieved using C3-DPPTT-T:PC_71_BM, which exceeded 8.8% despite a slightly wide band gap because of the high charge-carrier mobility.

Most research on alkyl side chains focused on improving solubility or optimising the molecular structure without causing special changes in the optical and electrical properties.^39–41^ However, Wang *et al.* reported oligo(ethylene glycol) (OEG) as a sidechain-substituted DPP-based polymer donor (PDPP3T-O16).^34^ The substitution of OEG as a side chain has been proven to effectively reduce the band gap because OEG can facilitate dense π–π intermolecular stacking of polymers while showing better solubility and flexibility than alkyl chains. Thus, PDPP3T-O16 exhibited not only a clearly narrower band gap (1.30 eV) but also a higher PCE (5.47%) than the alkyl-chain-substituted reference polymer (PDPP3T-C20, *E*_g_ = 1.34 eV, PCE = 3.00%).

Considering the latest DPP-based polymer donors, not only D–A-type polymer donors, such as the past DPP-based polymer donors, but also the complex molecular structure, terpolymer system, and D–A1–D–A2-type have been investigated. Furthermore, regarding device fabrication, it has been remarkable to manufacture OSCs by blending with NFAs, instead of fullerene derivatives, since the development of ITIC as an NFA in 2015.^42^ Janssen *et al.* reported α- and β-branched alkyl side chain-substituted DPP-based polymer donors (1′HD-PDPP3T and 2′HD-PDPP3T) to investigate the effect of side chains on the structural and optoelectronic properties.^43^ 1′HD-PDPP3T showed a relatively wide band gap (1.50 eV) because of the interruption of π–π intermolecular stacking caused by the α-branched alkyl side chain close to the conjugated backbone compared with 2′HD-PDPP3T (*E*_g_ = 1.32 eV). An OSC based on the polymer and PC_71_BM exhibited PCEs of 3.96% for 1′HD-PDPP3T and 7.10% for 2′HD-PDPP3T, respectively. This indicates that the optical properties of the polymer depend on the steric hindrance effect. Xia *et al.* studied polymer donors possessing the highest occupied molecular orbital (HOMO) energy level by changing their counterpart to realise a high open-circuit voltage (*V*_OC_).^44^ Thiothiophene-substituted benzo[1,2-*b*;4,5-*b*′]dithiophene (BDT-TS), which effectively adjusts the frontier molecular orbital energy levels to a deeper level, was selected as a counterpart because typical DPP-based polymers tend to possess high frontier, molecular orbital energy levels. PBDT-TS-DPP, comprising BDT-TS and DPP, showed a deeper HOMO energy level (−5.29 eV) and smaller *E*_g_ (1.38 eV) than PBDT-T-DPP, constituting a typical BDT (*E*_HOMO_ = −5.27 eV and *E*_g_ = 1.41 eV). The PCE of PBDT-TS-DPP:PC_71_BM was 5.48% with enhanced *V*_OC_, while the OSC based on PBDT-T-DPP:PC_71_BM exhibited a PCE of 4.78%. Nguyen *et al.* reported dithieno[3,2-*b*:2,3-*d*]pyrrole (DTP), which could reduce the optical band gap with high charge carrier mobility owing to the electron-rich N atom on DTP, and a polymer-based polymer donor (DPP-P3).^45^ DPP-P3 was found to have an absorption range up to 900 nm with *E*_g_ = 1.31 eV, and the corresponding fullerene-based OSC exhibited a maximum PCE of 4.85%.

Reynolds *et al.* reported a D–A-type polymer (P(T4-DPP-M)) with a methyl sidechain on the DPP unit, which displayed an absorption range up to 900 nm with the optical band gap of 1.40 eV.^46^ Furthermore, P(T4-DPP-M) exhibited competitive hole mobility values in the order of 10^−3^ cm^2^ V^−1^ S^−1^, realized from a space charge-limited current mobility experiment; this corresponded with the introduction of methyl side chains to reduce sterically hindered sites and enhance intermolecular interactions. An OSC based on P(T4-DPP-M):PC_71_BM was manufactured to investigate the photovoltaic performance and exhibited a maximum PCE of 5.4% with a high *J*_SC_ of 16.4 mA^2^ cm^−2^. Interestingly, ITIC was also selected as an NFA instead of PC_71_BM to utilise both the broad absorption profiles up to the NIR region for high *J*_SC_ and the high LUMO energy level for high *V*_OC_ of ITIC. However, P(T4-DPP-M):ITIC showed a low PCE of 2.2% with *J*_SC_ = 6.7 mA^2^ cm^−2^ despite an improved *V*_OC_ value (from 0.56 to 0.69 V), as expected. Recently, Durrant and Lee reported visibly transparent OSCs based on an NIR-harvesting bulk heterojunction blend.^47^ The visibly transparent OSC could selectively harvest NIR photons using a DPP-based polymer (DPP2T) as a donor and IEICO-4F as an NFA. This OSC based on DPP2T:IEICO-4F exhibited the highest PCE of 9.13% with a strong external quantum efficiency (EQE) response in the wavelength range of 700–900 nm. The visibly transparent OSC was manufactured by incorporating ultrathin Ag and MoO_3_ layers as transparent top electrode and antireflection layers, respectively, and exhibited a PCE of 5.74% with the visible transmittance reaching 60%. Notably, both opaque and visibly transparent OSCs displayed excellent air and photostable properties without a specific burn-in loss. Moreover, asymmetric heteroarene-flanked DPP is a complex molecular structure in the DPP unit, and various corresponding polymers have been reported since 2016.^48^ Janssen *et al.* developed two asymmetric thiophene/pyridine-flanked DPP-based polymers to compare the optical, electrochemical, and photovoltaic properties of region-random and region-regular conjugated backbones.^49^ The synthesized region-regular asymmetric polymer (P(TDPPPy-T)-*reg*) showed a stronger vibrionic peak and narrower band gap (1.49 eV) than the random polymer (P(TDPPPy-T)-*ran*, *E*_g_ = 1.53 eV). In addition, the OSC of P(TDPPPy-T)-*reg*:PC_71_BM exhibited a slightly higher PCE of 6.6% than that of P(TDPPy-T)-*ran*:PC_71_BM (PCE = 5.9%) because of the slightly increased *J*_SC_.

A terpolymer system containing three different monomers is effective for fine-tuning the optical and electrochemical properties as well as intermolecular packing.^50–54^ Typically, DPP-based polymers display weak absorption profiles in the wavelength range of 350–500 nm.^55–58^ By introducing fluorinated benzotriazole (FTAZ) as a secondary accepting building block, a ternary system was built into a copolymer system consisting of DPP and BDT to complement the weak absorption of short wavelengths.^59^ A strong absorption profile in the long-wavelength range of 600–900 nm (*E*_g_ = 1.43 eV) was observed for DPP-P4, which comprised DPP and FTAZ in the ratio of 3 : 1, while DPP-P5, comprising DPP : FTAZ of 1 : 1, showed a slightly blue-shifted absorption range and strong absorption in the wavelength range of 350–650 nm due to the weak ICT effect caused by the introduction of FTAZ. Fullerene-based OSCs were fabricated and exhibited PCEs of 5.58% and 5.24% for P1 and P2, respectively. In addition, non-fullerene-based OSCs comprising IEICO-4F as the NFA were fabricated and achieved PCEs of 5.24% and 5.49% for P1 and P2, respectively.

To further reduce the optical band gap, Cao *et al.* constructed a D–A1–D–A2-type polymer donor (PffBT-DPP) that not only exhibits a larger full width at half maximum (FWHM) but also enables finer modulation of the frontier molecular orbital energy levels, wherein A1 is the DPP, A2 is the benzothiadizole (BT) derivative, and D is thiophene. The D–A1–D–A2-type polymer donor enabled a strong ICT effect between the strong-withdrawing units (DPP and BT) and bithiophene, resulting in a broad FWHM and narrow optical bandgap (1.33 eV).^60^ Moreover, the estimated frontier molecular orbital energy levels of PffBT-DPP are −5.66 and −4.56 eV for the HOMO and LUMO, respectively, which enable a cascade energy level between PffBT-DPP and the electron acceptors (PC_71_BM and MeIC) for efficient OSCs. The OSC fabricated based on PffBT-DPP:PC_71_BM exhibited a PCE of 6.8%, whereas PffBT-DPP:MeIC achieved a rather poor PCE of 2.0%. This is because PffBT-DPP formed highly impure domains with MeIC, affecting the charge trap site. Interestingly, the ternary OSC based on PffBT-DPP:PC_71_BM:MeIC achieved an improved PCE (9.0%). This demonstrates the significance of the detailed miscibility between the donor and acceptor in establishing structure–performance relationships in OSCs. Huang *et al.* reported other D–A1–D–A2-type polymer donors.^61^ BT derivatives were also selected as secondary acceptor units to produce three polymer donors, TBT-DPP, FTBT-DPP, and HFTBT-DPP. These polymers also show a broad FWHM with a narrow optical band gap (<1.35 eV). However, PC_71_BM-based OSCs demonstrate a low PCE (<1.15%) despite realising a narrow band gap through state-of-the-art molecular design concepts. Shimizu *et al.* reported UNBG DPP-based polymer donors comprising pyrrolopyrrole aza-BODIPY (PPAB) that enabled NIR absorption because of the pi-extended structure.^62^ PPAB was polymerized with cyclopentadithiophene (CPDT), which is a strong electron-donating building block, to produce DPP-P6 and DPP-P8. DPP-P7 was produced using a terpolymer system comprising DPP, PPAB, and CPDT. As expected, polymers comprising PPAB absorbed up to 1200 nm with UNBGs of 1.08 eV for DPP-P6 and 1.12 eV for DPP-P7, respectively. The fullerene-based OSCs showed PCEs of 2.27%, 1.56%, and 1.23% for DPP-P6, DPP-P7, and DPP-P8, respectively.

### 2.3 BT-based narrow-band gap polymers

**Fig. 6:**
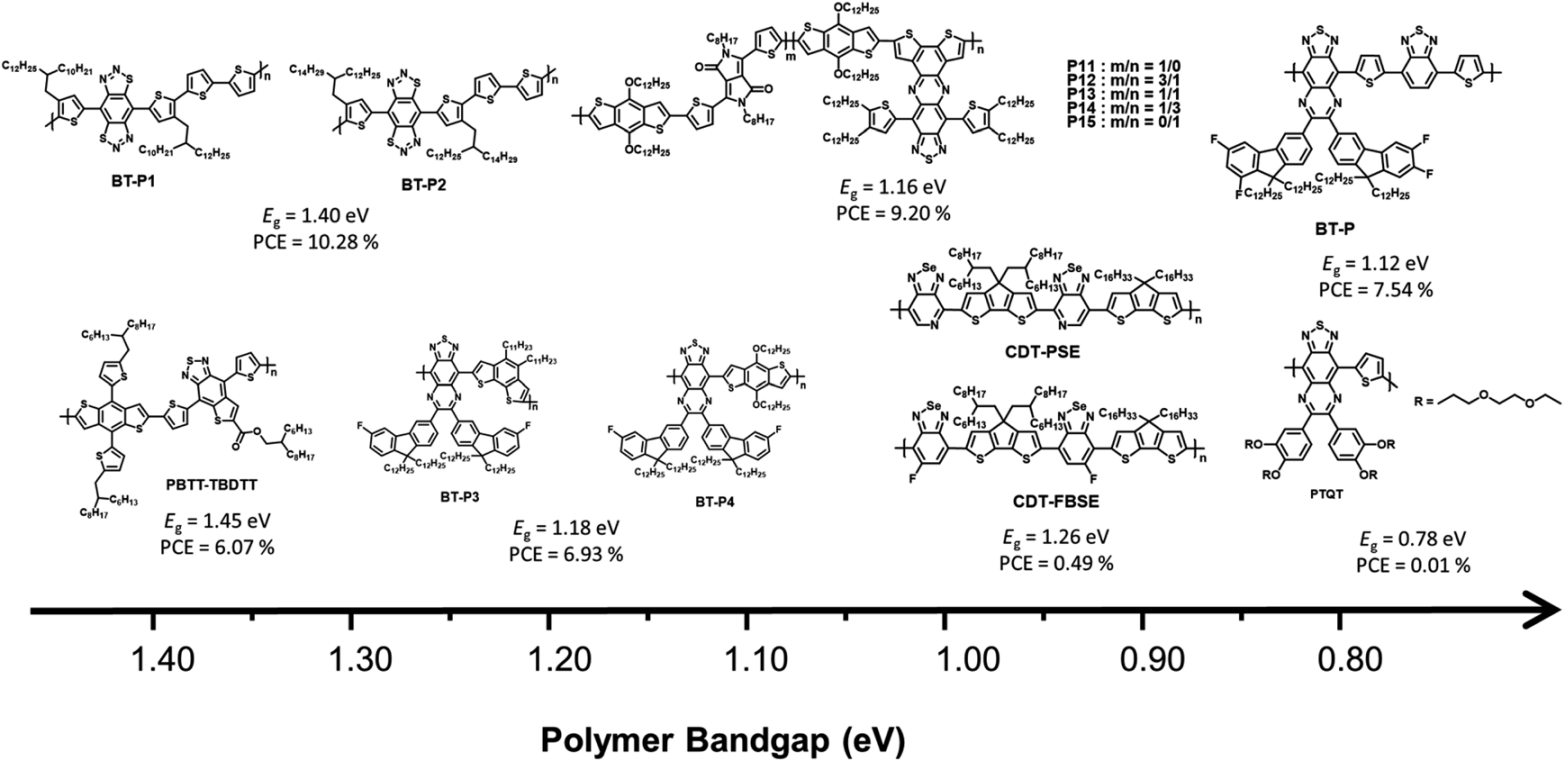
Chemical structures of benzothiadiazole-based polymers.

BT derivatives are strong electron-withdrawing building blocks that are used to construct NIR-absorbing materials.^63–66^ In particular, a fused molecular structure based on BT that can stabilize a quinoidal resonance form is more effective in narrowing the band gap than the commonly used BT derivative.^20,21^ This section describes the investigation of NIR-absorbing polymer donors based on BT derivatives. The molecular structure of the BT derivative, which depends on the optical band gap, is shown in Fig. 6, and the corresponding optical, electrochemical, and photovoltaic performances of the polymer donors are summarized in Table 3.

**Table 3:** Summary of the optical, electrochemical, and photovoltaic performances of BT-based polymers

Materials	*E* ^opt^_g_	*E*_LUMO_/*E*_HOMO_	Acceptor	*V*_OC_ (V)	*J*_SC_ (mA cm^−2^)	FF (%)	PCE (%)	*μ*_h_ [cm^2^ V^−1^ s^−1^]	*μ*_e_ [cm^2^ V^−1^ s^−1^]	Ref.
PBTT-TBDTT	1.45	−3.39/−5.35	PC_71_BM	0.75	13.15	0.63	6.07	8.89 × 10^−5^	—	67
BT-P1	1.40	−4.03/−5.43	PC_61_BM	0.81	20.83	0.61	10.28	2.1 × 10^−4^	1.8 × 10^−4^	68
BT-P2	1.37	−4.01/−5.38	PC_61_BM	0.83	14.09	0.64	7.52	1.6 × 10^−4^	1.4 × 10^−4^	
PTQT	0.78	−3.77/−4.55	PC_71_BM	0.80	0.18	0.19	0.01	—	—	71
BT-P3	1.06	−3.92/−5.38	PC_71_BM	0.72	9.66	0.58	4.03	8.05 × 10^−4^	2.56 × 10^−4^	73
BT-P4	1.18	−3.76/−5.26	PC_71_BM	0.69	14.34	0.70	6.93	1.18 × 10^−4^	2.45 × 10^−4^	
P	1.12	−3.83/−5.25	PC_71_BM	0.65	16.12	0.72	7.54	1.45 × 10^−4^	2.63 × 10^−4^	74
P11	1.34	−3.60/−5.23	PC_71_BM	0.83	13.88	0.64	7.37	8.4 × 10^−5^	2.31 × 10^−4^	72
P12	1.30	−3.63/−5.24	PC_71_BM	0.83	14.36	0.66	7.87	1.34 × 10^−4^	2.43 × 10^−4^	
P13	1.16	−3.67/−5.26	PC_71_BM	0.86	15.74	0.68	9.20	1.98 × 10^−4^	2.49	
P14	1.15	−3.70/−5.28	PC_71_BM	0.88	14.78	0.66	8.58	1.16 × 10^−4^	2.42 × 10^−4^	
P15	1.14	−3.73/−5.30	PC_71_BM	0.90	0.64	14.08	8.11	7.2 × 10^−5^	2.26 × 10^−4^	
CDT-PSE	1.02	−3.92/−4.94	PC_61_BM	0.53	1.45	0.30	0.24	—	—	75
CDT-FBSE	1.26	−3.91/−5.17	PC_61_BM	0.63	2.37	0.33	0.49	—	—	

In 2014, Qin *et al.* reported a novel strong electron-accepting building block, established by fusing a thiophene ring on BT, as thieno[2,3-*f*]-2,1,3-benzothiadiazole-6-carboxylate (BTT). BDT was polymerized with BTT *via* typical Stille polymerization to obtain an NIR-absorbing polymer donor (PBTT-TBDTT) for comparison with conventional BT-based reference polymer donors (PBT-TBDTT).^67^ The introduction of a carboxyl group on BTT is key to both stabilizing the quinoidal resonance form and reducing the HOMO energy level. Noticeably, PBTT-TBDTT showed a broad absorption range with a narrow optical band gap (1.43 eV) and a low-lying HOMO energy level (−5.35 eV), while the *E*_g_ of PBT-TBDTT was 1.70 eV. Consequently, the maximum PCE of PBTT-TBDTT:PC_71_BM was 6.07% with simultaneously enhanced *J*_SC_ and *V*_OC_ owing to the broad absorption range and deep HOMO energy level. Facchetti *et al.* developed NIR-absorbing polymer donors substituted with different alkyl sidechains (BT-P1 and BT-P2) based on benzo[1,2-*d*:4,5-*d*]bis([1,2,3]thiadiazole) (iso-BBT).^68^ The design strategy was inspired by both the regioisomer of benzobis[1,2-*c*;4,5-*c*]bis[1,2,5]thiadiazole (BBT) that is a strong nonclassical pi-electron-accepting building block and their prior research on the development of benzo[*d*][1,2,3]thiadiazole (iso-BT) as a well-known BT derivative.^69,70^ From the absorption onset of the film state, the optical band gaps were 1.40 eV for BT-P1 and 1.37 eV for BT-P2. By considering PC_61_BM as an electron acceptor, the OSCs displayed PCEs of 10.28% and 7.52% for BT-P1 and BT-P2, respectively, with a remarkable *E*_LOSS_ of <0.6 eV. Charas *et al.* reported a UNBG polymer donor (PTQT) containing thiophene and a thiadiazoloquinoxaline derivative.^71^ Although a UNBG (0.78 eV) was successfully achieved, the estimated HOMO energy level of PTQT was significantly high (−4.55 eV). The OSC based on PTQT and PC_71_BM had an inferior PCE of 0.01% because pronounced PC_71_BM aggregations that affect exciton dissociation and charge carrier transport were unfavourably affected by PTQT:PC_71_BM. Sharma *et al.* reported benzothiadiazole quinoxaline (BTQx) derivatives.^72,73^ The strong electron-accepting building block BTQx, in which the Qx derivative is fused onto the BT, is a suitable candidate to narrow the band gap because it can easily induce a stabilized quinoidal resonance form. Among the BTQx derivatives, fluorene-substituted BTQx and the corresponding polymers (BT-P3 and BT-P4) were designed and developed. As expected, both polymers showed broad absorption profiles with significantly narrow band gaps; in particular, BT-P3, comprising a strong donating benzo[2,1-*b*:3,4-*b*′]dithiophene, absorbed a broad range up to 1170 nm (*E*_g_ = 1.06 eV). OSCs based on the synthesized polymer and PC_71_BM were fabricated and exhibited maximum PCEs of 4.03% for BT-P3 and 6.93% for BT-P4. From the EQE curves, relatively low responses with valleys of 400–900 nm were observed in both OSCs. Although the OSCs were able to harvest broad sunlight below 1200 nm, the low *J*_SC_ values were correlated to the valleys from the EQE curves.

To finetune the optical and electrochemical properties as well as intermolecular packing, a D–A1–D–A2-type polymer donor was produced, comprising a thiophene donating building block and two different acceptors fluorinated BTQx and BT.^74^ The synthesized polymer (P), which enabled a strong ICT effect due to the introduction of BT, exhibited a broad absorption profile up to 1100 nm with the optical band gap of 1.12 eV. The OSC based on P:PC_71_BM showed a PCE of 7.54% after solvent addition followed by thermal annealing. However, the valley remained in the EQE curve. To address this problem, new D–A1–D–A2-type polymer donors using two kinds of accepting building blocks, DPP and BTQx, were developed to modulate the optical band gap and their photovoltaic properties.^72^ Considering P11–P15, P15, containing DPP:BTQx contents of 0 : 100, displayed the narrowest optical band gap at 1.14 eV. With increasing DPP content, the optical band gaps of the polymer donor gradually increased from 1.15 to 1.30 eV. In addition, P11 without BTQx demonstrated a narrow absorption range and broad band gap of 1.34 eV. The fabricated OSC based on P15:PC_71_BM showed a PCE of 8.11% despite the valley from 450 to 900 nm in the EQE curve, while the PCE of BT-P11:PC_71_BM was 7.37%. Among the D–A1–D–A2-type polymers, the OSC based on BT-P13:PC_71_BM exhibited the highest PCE of 9.20% and a broad EQE response up to 1050 nm without a valley appearing in the EQE curve. This demonstrates that the D–A1–D–A2-type polymer can complement the disadvantages of each acceptor, including the absorption range or frontier molecular orbital energy levels. Liu *et al.* reported a D1–A–D2–A-type polymer donor containing [1,2,5]selenadiazole[3,4-*c*]pyridine (PSe) with a photocurrent response of up to 1100 nm.^75^ CPDT derivatives with different alkyl side chains (D1 and D2) were selected as strong electron-donating building blocks to overcome the poor solubility of PSe. The D1–A–D2–A-type regular polymer based on PSe (CDT-PSE) displayed a significantly broad absorption range up to 1200 nm with the optical band gap of 1.02 eV whereas, a relatively wide band gap was observed in the CDT-FBSE film containing 5-fluorobenzo[*c*][1,2,5]selenadiazole (FBSe) due to the weak ICT effect between CPDT and FBSe. Although the optimum OSC based on PC_61_BM displayed low PCEs of 0.49% for CDT-FBSE and 0.24% for CDT-PSE, the broad photoresponse wavelength over 1000 nm results in great potential for NIR applications.

### 2.4 Isoindigo-based narrow-band gap polymers

**Fig. 7:**
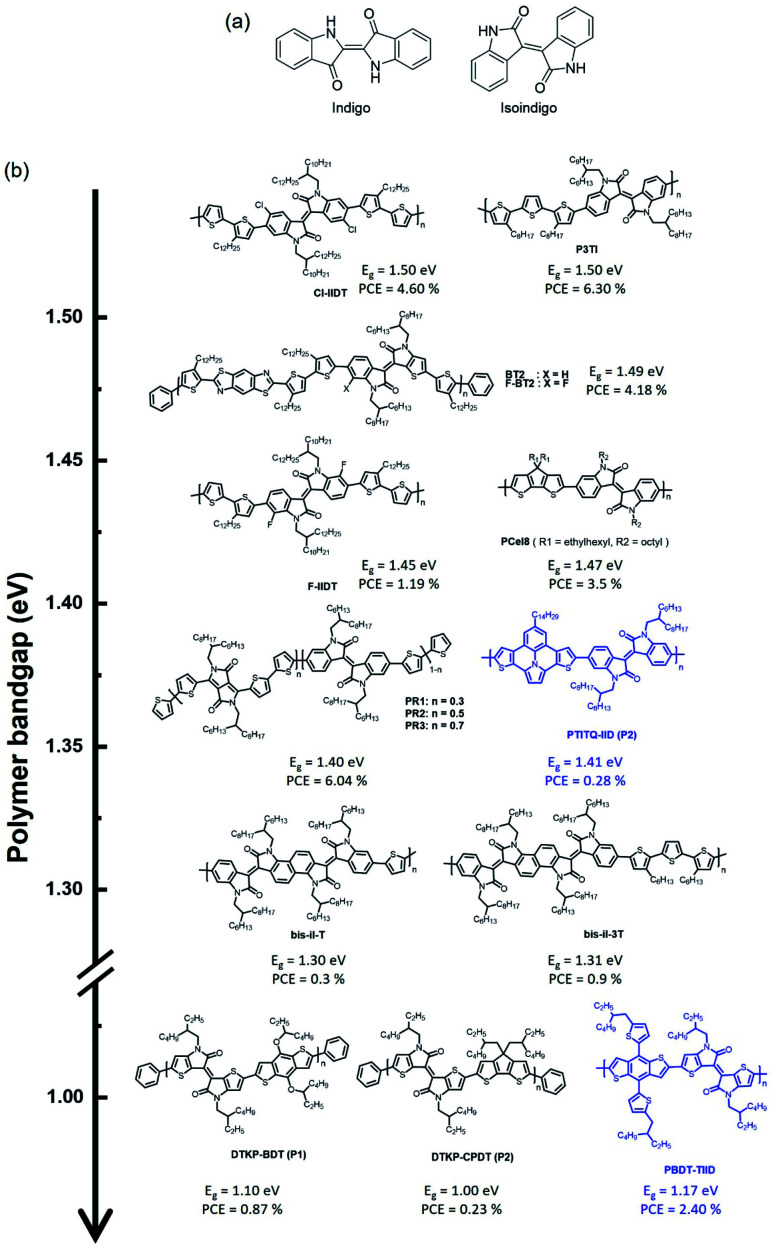
(a) Chemical structure of indigo, isoindigo and (b) isoindigo-based polymers.

The chemical structure of isoindigo contains two five-membered lactam rings joined by an exocyclic double bond at the 3 and 3′ positions. Isoindigo is a naturally occurring indigoid pigment, a geometric isomer of the common dye indigo, as shown in Fig. 7(a).^76,77^ Isoindigo has a planar p-conjugated symmetric structure originating from a fully conjugated structure and electron-withdrawing properties owing to its two lactam rings. After being synthesized and reported in 1988,^78^ it was only recognized in 2010 by Reynolds *et al.* that small molecules based on isoindigo have demonstrated the potential for use as electron donors in OSCs.^79^ In 2011, Wang *et al.* reported a narrow-bandgap polymer donor (P3TI, *E*_g_ = 1.5 eV) comprising isoindigo and terthiophene in the polymer backbone, and fullerene-based OSCs showed a PCE of 6.3%.^80^ Various isoindigo-based polymer donors have been reported and studied since the synthesis of P3TI.^81–84^ This section presents a brief summary of isoindigo-based polymer donors with an UNBG, and the molecular structure is shown in Fig. 7(b). The latest polymer donors are highlighted in blue. Additionally, the corresponding optical, electrochemical, and photovoltaic performances of the isoindigo-based polymer donors are summarized in Table 4.

**Table 4:** Summary of the optical, electrochemical, photovoltaic performances of isoindigo-based polymers

Materials	*E* ^opt^_g_ (eV)	*E*_LUMO_/*E*_HOMO_	Acceptor	*V*_OC_ (V)	*J*_SC_ (mA cm^−2^)	FF (%)	PCE (%)	*μ*_h_ [cm^2^ V^−1^ s^−1^]	*μ*_e_ [cm^2^ V^−1^ s^−1^]	Ref.
P3TI	1.50	−3.83/−5.82	PC_71_BM	0.70	13.00	0.69	6.30	—	—	80
Cl-IIDT	1.50	−3.85/−5.53	PC_71_BM	0.75	10.00	0.61	4.60	1.06 × 10^−5^	—	85
F-IIDT	1.45	−3.92/−5.51	PC_71_BM	0.63	4.26	0.44	1.19	2.07 × 10^−5^	—	
BT2	1.49	−3.92/−5.41	PC_71_BM	0.62	10.07	0.67	4.18	—	—	86
F-BT2	1.49	−3.95/−5.44	PC_71_BM	0.64	4.48	0.57	1.66	—	—	
PC8I8	1.37	−3.89/−5.24	PC_61_BM	0.66	10.10	0.5	3.30	—	—	87
PC8Ie	1.42	−3.88/−5.31	PC_61_BM	0.73	8.20	0.45	2.70	—	—	
PCeI8	1.47	−3.85/−5.33	PC_61_BM	0.79	9.70	0.46	3.60	—	—	
PCeIe	1.48	−3.96/−5.45	PC_61_BM	0.80	11.60	0.43	4.00	—	—	
PR1	1.46	−3.79/−5.50	PC_71_BM	0.78	7.84	0.57	3.49	—	—	88
PR2	1.40	−3.89/−5.62	PC_71_BM	0.77	13.52	0.58	6.04	—	—	
PR3	1.38	−3.92/−5.63	PC_71_BM	0.69	12.35	0.57	4.86	—	—	
PTITQ-IID	1.41	−3.58/−4.98	PC_61_BM	0.59	1.24	0.47	0.34			89
Bis-il-T	1.30	−4.09/−5.73	PC_71_BM	0.80	0.89	0.59	0.42	—	—	90
Bis-il-3T	1.31	−4.02/−5.64	PC_71_BM	0.66	2.61	0.62	1.07	—	—	
DTKP-BDT	1.10	−3.80/−4.90	PCBM	0.40	4.75	0.46	0.87	—	—	91
DTKP-CPDT	1.00	−3.80/−4.80	PCBM	0.31	1.23	0.59	0.23	—	—	
PBDT-TIID	1.17	−3.68/−5.06	PC_71_BM	0.42	10.34	0.54	2.40	1.75 × 10^−4^	6.72 × 10^−4^	92

One method for narrowing the band gap involves the introduction of halogens with electron-withdrawing properties, such as fluorine, bromine, and chlorine, onto the polymer backbone, which is an effective strategy for structure manipulation to finely tune energy levels and phase separation simultaneously. By introducing halogens onto the polymer backbone, two isoindigo polymers, substituted with chlorine (Cl-IIDT) and fluorine (F-IIDT), were synthesised by Zheng *et al.* in 2015.^85^ The incorporation of electron-withdrawing halogens on the polymer backbone can effectively induce low-lying frontier molecular orbital energy levels and expand the absorption spectra to longer wavelengths. As a result, Cl-IIDT containing chlorine, which has a stronger electron affinity than fluorine, showed a low-lying LUMO energy level and a high-lying HOMO energy level, resulting in a narrower optical band gap than F-IIDT. Moreover, planarity of the polymer backbone was improved by halogen substitutions because the lone pair of a halogen can noncovalently interact with a neighbouring S atom of thiophene. As a result, the OSC based on Cl-IIDT and PC_71_BM showed a PCE of 4.60%, while that based on F-IIDT:PC_71_BM exhibited a low PCE (1.19%) because of the unfavourable film morphology, which was due to the high crystallization tendency of F-IIDT, observed in F-IIDT:PC_71_BM. Contrary to F-IIDT, Cl-IIDT, which contains Cl with a larger atomic size than F, can generate a relatively distorted polymer backbone to reduce interchain interactions. In addition, Ide *et al.* reported a new conjugated polymer constituting fluorinated benzothienoisoindigo (F-BTIDG) and nonfluorinated F-BTIDG.^86^ F-BT2 containing F-BTIDG exhibited a low PCE of 1.66% despite the planar geometric structure by introducing fluorine whereas, the PCE of BT2:PC71BM reached 4.18% owing to the preference of face-on orientation for beneficial charge transfer in the OSC.

An appropriate choice of electron-donating building block is essential to develop an ideal and sustainably low band gap. Ho *et al.* reported four types of narrow-bandga p isoindigo-based polymer donors containing CPDT as an electron-donating building block.^87^ They explained that specific electron-donating and electron-accepting building blocks were chosen because these monomers (CPDT and isoindigo) are able to satisfy the requirements a strong acceptor and a weak donor, leading to a strong ICT effect. The synthesized polymers exhibited broad absorption bands and optical band gaps at 1.37–1.48 eV. Furthermore, the steric hindrance effects of bulky alkyl side chains in PCeIe caused a reduction in the ICT effect, and thus increased their optical band gaps. They also argued that by increasing the steric hindrance, the HOMO energy level can be lowered, while the LUMO energy levels are less affected.

Jung *et al.* reported random copolymers with two kinds of electron-accepting building blocks, DPP and isoindigo, demonstrating complementary absorption in D–A-type polymer donors for NIR-absorbing OSCs.^88^ The copolymer PIT comprises thiophene as an electron-donating building block and isoindigo-absorbed photons shorter than 750 nm in wavelength (*E*_g_ = 1.63 eV) with a low-lying HOMO energy level of around −5.65 eV. Meanwhile, the DPP-based polymer donor (PDPP3T) exhibited NIR absorption over 900 nm with a relatively high-lying HOMO energy level around −5.35 eV compared with that of PIT. The random copolymers absorbed a wide range of solar spectra from 600 to 900 nm and possessed low-lying HOMO energy levels. In particular, PR2 (DPP/isoindigo = 0.5/0.5) showed ideal absorption profiles with a broad FWHM, as mentioned above, and appropriate frontier molecular orbital energy levels for achieving high-performance OSCs. Consequently, PR2:PC_71_BM demonstrated the best PCE of 6.04% with a high *J*_SC_ value, whereas low PCEs were measured in PIT:PC_71_BM and DPP3T:PC_71_BM. Recently, Miao *et al.* used 7-tetradecylthieno[3′,2′:7,8]indolizino[6,5,4,3-*ija*]thieno[2,3-*c*]quinolone as an electron-donating building block, which is expected to have a strong ICT effect to induce a narrow band gap, that was polymerized with isoindigo *via* commercial Stille polymerization to obtain PTITQ-IID.^89^ As expected, PTITQ-IID showed a broad absorption range up to 900 nm, and the HOMO/LUMO energy levels were estimated at −4.98 and −3.58 eV, respectively. However, the corresponding OSC exhibited an inferior PCE of 0.28% because of the unfavourable morphology, including aggregated regions, for the OSC. Moreover, Randell *et al.* developed isoindigo dimer (di-iI) and bisisoindigo (bis-iI) to investigate the effects of the acceptor unit length and planarity on the optoelectronic properties.^90^ The coplanarity of polymer donors (bis-iI-T and bis-iI-T) containing bis-iI substantially reduced the optical band gap to around 1.62 eV relative to those of isoindigo- and di-iI-based polymer donors. They found that bis-iI-based polymer donors displayed the trends of decreasing LUMO energy levels and increasing electron mobility. However, although bis-iI-based polymer donors absorbed well up to 950 nm, negligible photocurrents were produced beyond 700 nm. They demonstrated that the effects of the acceptor unit length and planarity provide an effective approach for finetuning the optical and electrochemical properties of OCSs.

To further reduce the optical band gap, thienoisoindigo (TII), produced by replacing the outer benzene of isoindigo with thiophene, has been introduced, which is expected to improve planarity along the polymer backbone, resulting in strong stacking between molecules and improved charge carrier mobilities.^91^ Koizumi *et al.* reported various TII-based polymer donors that produced extensively narrow band gaps up to 1.00 eV compared with typical isoindigo-based polymer donors.^93^ TIID is a promising candidate as an electron-accepting building block for NIR-absorbing OSCs. Wang *et al.* reported PBDTT-TIID comprising benzodithiophene and TIID in 2016.^92^ Owing to the strong electron-deficient and highly coplanar conformation of TIID, PBDT-TIID exhibited a very narrow band gap (1.17 eV) with a high-lying HOMO energy level of −5.06 eV. Although a narrow band gap was successfully realized, the optimum PCE of PBDTT-TIID:PC_71_BM was 2.40% with a significantly low *V*_OC_ (0.42 V) owing to the high-lying HOMO energy level of PBDT-TIID.

### 2.5 Other polymers

**Fig. 8:**
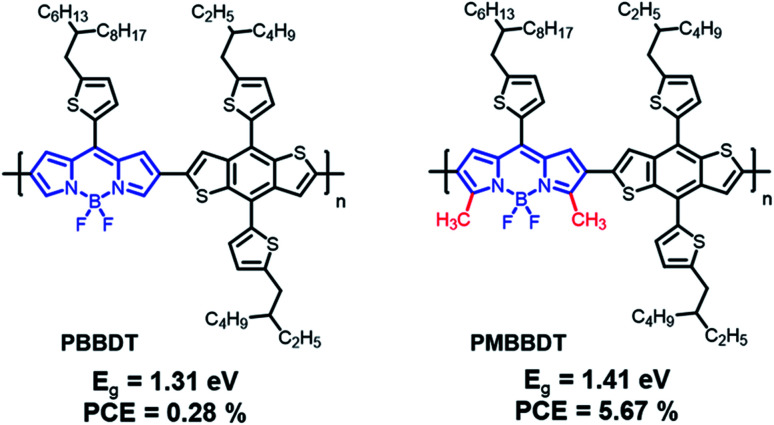
Chemical structures of boron dipyrromethene (BODIPY)-based polymers and B–N bonds on the benzothiadiazole polymer.

For the development of narrow-bandgap polymers, two types of repeating units that are well-known chemical structures have recently been reported, as shown in Fig. 8 and summarized in Table 5. First, Guo *et al.* reported boron dipyrromethene (BODIPY) as an electron-deficient unit that can also be used to construct D–A-type conjugated polymers that absorb in the NIR region.^94^ The PBBDT exhibited NIR absorption at around 900 nm with the optical band gap of 1.31 eV, whereas PMBBDT, formed by substituting two methyl units in BODIPY, showed slightly blue-shifted absorption profiles because the substituted methyl units act as weak-donating units, resulting in a reduction in the electron-accepting properties. In this study, N2200 was used as an electron acceptor, and the OSC based on PMBBDT:N2200 exhibited the best PCE of 5.8% with a broad photoresponse from 300 to 900 nm owing to improved hole mobility by introducing methyl, despite the slightly blue-shifted absorption profile; however, PBBDT:N2220 showed a PCE of 0.32%. Recently, PMBBDT with excellent potential, including broad absorption and high charge carrier mobility for NIR-absorbing OSCs, was investigated further for application in non-fullerene-based OCSs.^95^ Two chlorinated electron acceptors, ITIC-2Cl and BTP-2Cl, were carefully selected to satisfy the cascade stage between the electron donor and acceptor. The fabricated OSC of PMBBDT:ITIC-2Cl displayed a maximum PCE of 7.56% due to the limited *J*_SC_ of 17.4 mA cm^−2^, while PMBBDT:BTP-2Cl achieved the best PCE of 9.86% with *J*_SC_ = 21.44 mA cm^−2^. It seems that the broad absorption range and FWHM of BTP-2Cl contribute greatly to improving *J*_SC_. Notably, the measured *J*_SC_ value of PMBBDT-BTP-2Cl (21.44 mA cm^−2^) is the highest photocurrent among OSCs based on polymer donors containing BODIPY. These results demonstrate that BODIPY-based conjugated polymers are promising candidates for NIR-absorbing OSCs and suggest a new paradigm that doesn’t involve traditional molecular design concepts.

**Table 5:** Summary of the optical, electrochemical, and photovoltaic performances of boron dipyrromethene (BODIPY)-based polymer donors

Materials	*E* ^opt^_g_ (eV)	*E*_LUMO_/*E*_HOMO_	Acceptor	*V*_OC_ (V)	*J*_SC_ (mA cm^−2^)	FF (%)	PCE (%)	*μ*_h_ [cm^2^ V^−1^ s^−1^]	*μ*_e_ [cm^2^ V^−1^ s^−1^]	Ref.
PBBDT	1.31	−4.05/−5.36	N2200	0.69	1.52	0.31	0.32	2.0 × 10^−7^		94
PMBBDT	1.41	−3.90/−5.31	N2200	0.83	13.9	0.5	5.8	1.9 × 10^−3^		95
			ITIC-2Cl	0.72	17.40	0.60	7.56	1.12 × 10^−3^	1.15 × 10^−4^	
			BTP-2Cl	0.77	21.44	0.60	9.86	1.42 × 10^−3^	2.47 × 10^−4^	

## 3 Summary and outlook

For the structural design of UNBG, strong intra/inter-molecular charge transfer strategies are usually used to control the band gap in the D–A-type polymer backbone. The band gap-narrowing effects are dependent on the strength of the “A” unit (electron accepting or withdrawing units), and representative “A” groups including DPP, BT, and isoindigo. In this review, the dependence of the optical band gap of UNBG polymer donors was examined according to the type of electron-withdrawing groups. Thus far, with band gaps larger than 1.3 eV, many polymer donors comprising DPP, typical-BT, or isoindigo and exhibiting moderate PCEs have been reported. However, UNBG polymers (below 1.0 eV) showing high photovoltaic performances have seldom been reported despite the successful realization of UNBG through introduction of strong withdrawing groups (thienoisoindigo or fused-BT). Because the smaller the energy bandgap, the more difficult the HOMO/LUMO energy matching between the electron donor and acceptor in this narrow-band gap system, a systematic study of the driving force of exciton dissociation is also required. Moreover, most NIR-absorbing OSCs have been fabricated with PCBM until now; in particular, only a few OSCs comprise state-of-the-art NFAs. Consequently, research on OSCs comprising NIR polymer donors and NFAs should be conducted continuously, and the development of state-of-the-art strong electron-withdrawing units to solve the mismatch between the HOMO/LUMO energy levels of the electron donor and acceptor is necessary. For example, boron dipyrromethene (BODIPY) with B–N bonds has not yet been studied, and only a few groups have synthesized and characterized BODIPY-based polymer donors. Although the absolute photovoltaic performances of BODIPY-based polymers are inferior to those of traditional polymer donors such as DPP, BT, and isoindigo, BODIPY-based polymers are promising candidates for NIR-absorbing OSCs because they exhibit sufficient potential, and are able to avoid conventional molecular design concepts. As a result, when this narrow band gap polymer is developed, it can be used for two different purposes: first, with the narrow band gap acceptor, it will lead to the realization of transparent energy harvesting applications. In addition, with the wide band gap acceptor, the absorption wavelength range does not overlap, resulting in panchromatic absorption for efficient photocurrent gain. Thus, for next generation energy material development, we believe that the design and synthesis of novel narrow band gap polymer structures as well as a deeper fundamental understanding of the physics and chemistry of the molecules are required.

## Conflicts of interest

There are no conflicts to declare.

## Supplementary Material
